# Sex-steroid hormones and risk of postmenopausal estrogen receptor-positive breast cancer: a case-cohort analysis

**DOI:** 10.21203/rs.3.rs-3406466/v1

**Published:** 2023-10-09

**Authors:** Frances EM Albers, Makayla WC Lou, S Ghazaleh Dashti, Christopher TV Swain, Sabina Rinaldi, Vivian Viallon, Amalia Karahalios, Kristy A Brown, Marc J Gunter, Roger L Milne, Dallas R English, Brigid M Lynch

**Affiliations:** University of Melbourne; University of Melbourne; Murdoch Children’s Research Institute; University of Melbourne; International Agency for Research on Cancer; International Agency for Research on Cancer; University of Melbourne; University of Kansas Medical Center; Imperial College London; Cancer Council Victoria; Cancer Council Victoria; Cancer Council Victoria

**Keywords:** breast cancer, sex-steroid hormones, progesterone, estrogens, androgens, sex hormone binding globulin

## Abstract

**Purpose:**

Sex-steroid hormones are associated with postmenopausal breast cancer but potential confounding from other biological pathways is rarely considered. We estimated risk ratios for sex-steroid hormone biomarkers in relation to postmenopausal estrogen receptor (ER)-positive breast cancer, while accounting for biomarkers from insulin/insulin-like growth factor-signaling and inflammatory pathways.

**Methods:**

This analysis included 1,208 women from a case-cohort study of postmenopausal breast cancer within the Melbourne Collaborative Cohort Study. Weighted Poisson regression with a robust variance estimator was used to estimate risk ratios (RRs) and 95% confidence intervals (CIs) of postmenopausal ER-positive breast cancer, per doubling plasma concentration of progesterone, estrogens, androgens, and sex hormone binding globulin (SHBG). Analyses included sociodemographic and lifestyle confounders, and other biomarkers identified as potential confounders.

**Results:**

Increased risks of postmenopausal ER-positive breast cancer were observed per doubling plasma concentration of progesterone (RR: 1.22, 95% CI: 1.03 to 1.44), androstenedione (RR: 1.20, 95% CI: 0.99 to 1.45), dehydroepiandrosterone (RR: 1.15, 95% CI: 1.00 to 1.34), total testosterone (RR: 1.11, 95% CI: 0.96 to 1.29), free testosterone (RR: 1.12, 95% CI: 0.98 to 1.28), estrone (RR: 1.21, 95% CI: 0.99 to 1.48), total estradiol (RR: 1.19, 95% CI: 1.02 to 1.39) and free estradiol (RR: 1.22, 95% CI: 1.05 to 1.41). A possible decreased risk was observed for SHBG (RR: 0.83, 95% CI: 0.66 to 1.05).

**Conclusion:**

Progesterone, estrogens and androgens likely increase postmenopausal ER-positive breast cancer risk, whereas SHBG may decrease risk. These findings strengthen the causal evidence surrounding the sex hormone-driven nature of postmenopausal breast cancer.

## 1. Background

Breast cancer is a largely hormone-driven disease and the relationships between endogenous sex-steroid hormones – especially estrogens – and postmenopausal breast cancer are thought to be well established [1–3]. A recent systematic review and meta-analysis found moderate- to high-quality evidence that higher levels of estrogens (estradiol and estrone) and androgens (testosterone and androstenedione), and lower levels of sex hormone binding globulin (SHBG), were associated with increased risks of postmenopausal breast cancer [4]. Dose-response relationships were observed for SHBG, estradiol, and estrone, with weaker evidence for androstenedione and testosterone [4]. There was also evidence to suggest that progesterone and dehydroepiandrosterone (DHEA) were not associated with breast cancer [4].

The quality of the evidence in this review was largely determined by dose-response effects and large effect sizes [4]. No extracted result had adjusted for biomarkers from other biological pathways; namely, the insulin/insulin-like growth factor (IGF)-signaling and inflammatory pathways. These pathways may confound the effect of the sex-steroid hormone pathway. For example, insulin and insulin-like growth factor-1 (IGF-1) can affect the bioavailability of estrogens and androgens via the regulation of aromatase and suppression of hepatic SHBG production [1, 5, 6]. They may also play a role in breast carcinogenesis: insulin and the IGF axis are proposed to have mitogenic and anti-apoptotic properties, and higher systemic concentrations of IGF-1 are associated with increased risks of breast cancer [1, 2, 5–8]. Further, a state of low-grade chronic inflammation – for example, in the context of physical inactivity and obesity – can foster a pro-carcinogenic environment via the overstimulation and dysregulation of immune cells, cytokines and adipokines [1, 2, 5, 6, 9]. Higher circulating levels of C-reactive protein (CRP) – a non-specific marker of chronic inflammation – are associated with increased risks of breast cancer, but the epidemiological evidence for other inflammatory markers remains uncertain [2, 10, 11]. Higher circulating levels of pro-inflammatory biomarkers including leptin, tumor necrosis factor-alpha (TNF-α) and interleukin-6 (IL-6) are also associated with enhanced aromatase activity and lower circulating levels of SHBG [1, 2, 5, 6, 9, 12].

Studies that adjust for other biomarkers typically compare results with and without adjustment for other sex-steroid hormones and/or SHBG [3, 13–22]. These are often mutual or progressive adjustments to assess independence rather than confounding, on the basis that biomarkers share complex interrelationships and correlations. However, this practice can lead to overadjustment bias [23]. In addition, only a handful of studies have measured and adjusted for biomarkers from other biological pathways that may be potential confounders. One study from the Women’s Health Initiative presented results for estradiol with and without adjustment for free IGF-1 and insulin; positive associations with postmenopausal breast cancer appeared stronger with adjustment for both IGF-1 and insulin [24]. Another study from the UK Biobank presented results for testosterone with and without adjustment for SHBG and IGF-1 that were not appreciably different [25]. Further studies are needed to clarify the confounding role of other biological pathways implicated in breast carcinogenesis.

The aim of this study was to estimate risk ratios for sex-steroid hormone biomarkers in relation to postmenopausal breast cancer in a case-cohort of postmenopausal women within the Melbourne Collaborative Cohort Study (MCCS), while accounting for other biomarkers from the insulin/IGF-signaling and inflammatory pathways.

## 2. Methods

### 2.1. The Melbourne Collaborative Cohort Study

The MCCS includes 24,469 women aged 40–69 at recruitment from 1990–1994 [26]. At baseline and the second follow-up (F2, 2003–7), participants provided information about health status, lifestyle factors, sociodemographics and medical history via structured questionnaires [26]. Anthropometric and clinical measurements were performed at the study center, including the collection of blood samples [26]. At both times, plasma was stored in liquid nitrogen. Data linkages to national and state death and cancer registries – including the Victorian Cancer Registry and Australian Cancer Database – enabled vital status and cancer diagnoses to be determined prospectively [26]. The study protocol was approved by the Cancer Council Victoria Human Research Ethics Committee.

### 2.2. The case-cohort study

#### 2.2.1. Initial eligibility criteria at second follow-up (2003–7)

This case-cohort study was restricted to women who attended F2. At F2, eligible women were postmenopausal, not known to be taking hormone replacement therapy (HRT), had provided a blood sample (within one year of the F2 questionnaire, if completed), had no prior invasive cancer diagnosis (except for keratinocyte cancers); at baseline, they had a body mass index (BMI) ≥ 18.5 kg/m^2^. Women were considered postmenopausal if they had had no menstrual periods in the past 12 months and met one of the following criteria: had experienced natural cessation of menses; had a bilateral oophorectomy; were age 55 years or older; or had had no periods in the 12 months prior to baseline and, for participants in a previous case-cohort study, measured estradiol concentration below 109 pmol/L at baseline (a threshold from that study [13, 27]). The case-cohort comprised a random sample of the 10,669 eligible women and all eligible women diagnosed with estrogen receptor (ER)-positive postmenopausal breast cancer between blood collection at F2 and 31 October 2020.

An eligible tumor was defined as invasive adenocarcinoma of the breast (International Classification of Diseases, Tenth Revision [ICD-10] code C50) that was ER-positive. Tumors of unknown hormone receptor status were included as 88% of breast cancer diagnoses among eligible women of known ER status were ER-positive. ER-negative and progesterone receptor (PR)-positive cancers were also included as this tumor subtype may be misclassified and accounts for only 1–4% of diagnoses [28–31]. Unspecified adenocarcinomas and unspecified cancers were presumed to be adenocarcinomas as 99% of breast cancer diagnoses among eligible women of known morphology were adenocarcinomas.

**Figure 1.**
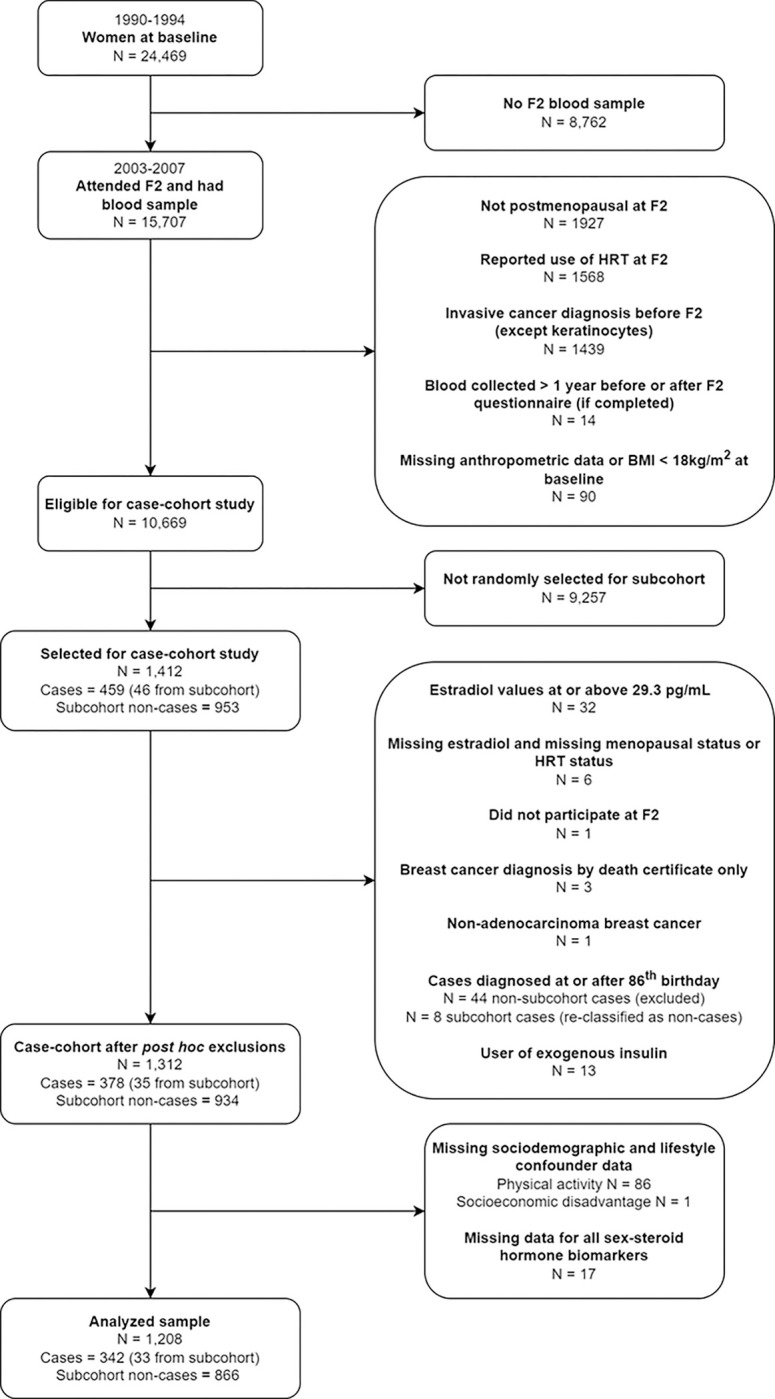
Selection of participants into the case-cohort study and analyses N: Number. F2: Second follow up wave. HRT: Hormone replacement therapy.

In total, 1,412 women were selected for the case-cohort study, including 999 in the subcohort and 459 cases (46 from the subcohort) (Fig. 1). The subcohort was a random sample of eligible women (Online Resource 1).

#### 2.2.2. *Post hoc* criteria

Of the 1,412 selected women, 286 (20%) had unknown menopausal status and/or HRT use. Eligibility was confirmed for all selected women using the distribution of measured estradiol values at F2 for naturally postmenopausal women who were not taking HRT (806, 57% of selected women). Thirty-two women with estradiol values at or above the 99th percentile of this distribution (29.3 pg/mL, equivalent to 107.6 pmol/L) were excluded, regardless of age, menopausal status, or HRT use. Menopausal status and/or HRT use could not be determined for six women missing estradiol measurements. One woman was excluded as she did not participate in F2 despite providing a blood sample.

Four cases outside the subcohort were retrospectively disqualified as cases; three diagnoses were ascertained from death certificate only and one woman was diagnosed with non-adenocarcinoma breast cancer. To minimize the impact of death as a competing risk, follow-up was chosen to end on participants’ 86th birthday (Online Resource 2). Thus, 44 cases outside the subcohort were excluded and eight cases within the subcohort were analyzed as non-cases. Thirteen users of exogenous insulin were excluded so that measured insulin concentrations were of endogenous insulin.

The total study sample after *post hoc* exclusions comprised 1,312 women, 969 in the subcohort and 378 cases (35 also in the subcohort) (Fig. 1).

### 2.3. Laboratory analysis of plasma biomarkers

Plasma samples of selected women were randomly ordered and allocated into 21 batches containing approximately equal numbers of cases. The samples were shipped on dry ice in two dispatches to the International Agency for Research on Cancer (IARC).

The plasma concentrations of all biomarkers were measured at the Nutrition Metabolism Branch, IARC. Plasma concentrations of sex-steroid hormones and SHBG were measured as previously described [32]. In brief, sex-steroid hormone concentrations were measured using a liquid chromatography-mass spectrometry system consisting of an ultra-high-performance liquid chromatograph (Agilent 1290, Agilent, Santa Clara, CA) and a QTRAP 5500 mass spectrometer (SCIEX, Framingham, MA). SHBG concentrations were measured by solid-phase “sandwich” enzyme-linked immunoassay (DRG International, Springfield, NJ). Interferon gamma (IFN-γ), IL-6, interleukin-8 (IL-8), interleukin-10 (IL-10), TNF-α, insulin, adiponectin, leptin, and CRP were measured by highly sensitive and highly specific electrochemiluminescent methods (Meso Scale Discovery, Rockville, MD). IGF-1 and insulin-like growth factor binding protein-3 (IGFBP-3) were measured by immunoassay methods by R&D Systems (Biotechne, Minneapolis, USA). C-peptide was measured by an enzyme-linked immunosorbent assay by ALPCO (Salem, USA). Further details are included in Online Resource 3. Three quality control samples at different concentration levels were measured in duplicate in each batch of analyses to assess the reliability of biomarker measurements. Reliability was assessed by calculating intra-assay and inter-assay coefficients of variation (CVs), as well as intra-batch and inter-batch intra-class correlation coefficients (ICCs), as described in Online Resource 4. Assay performance for estradiol and testosterone was evaluated by measuring samples created from reference standards with known concentrations. Measured values were compared with true values using validity coefficients and correlation plots, as described in Online Resource 5.

### 2.4. Normalization of biomarker values

Biomarker data were cleaned and normalized to correct for effects of batch, dispatch, and time since last meal (12% of study participants were not fasting at blood collection). The normalization technique was adapted from Viallon et al. [33]. Normalization models were used to estimate residual ICCs for the total proportion of variation attributable to batch for each biomarker. Methods for normalization and estimated ICCs are presented in Online Resource 6.

### 2.5. Calculation of free estradiol and free testosterone

Concentrations of free estradiol and free testosterone (i.e., not bound to SHBG) were calculated from normalized values of estradiol, testosterone and SHBG using the law of mass action assuming a fixed albumin concentration of 40 g/L (5.97 × 10^– 4^ mol/L) and the following association constants: 6 × 10^4^ L/mol (binding of estradiol to albumin); 4 × 10^4^ L/mol (binding of testosterone to albumin); 0.68 × 10^9^ L/mol (binding of estradiol to SHBG); 1.6 × 10^9^ L/mol (binding of testosterone to SHBG) [34–37].

### 2.6. Statistical analysis

Descriptive statistics were presented as medians and interquartile ranges (IQRs) or as frequencies and percentages, where appropriate. Weighted modified Poisson regression with a robust variance estimator was used to estimate risk ratios (RRs) and 95% confidence intervals (CIs) of postmenopausal ER-positive breast cancer, per doubling plasma concentration of progesterone, androstenedione, DHEA, total and calculated free testosterone, estrone, total and calculated free estradiol, and SHBG. Case weights were one, and weights for non-cases were the inverse of the sampling probability for non-cases [38].

Confounders including other biomarkers were identified *a priori* using causal diagrams informed by expert consensus and literature review. Sociodemographic and lifestyle confounders included: education; country of birth; socioeconomic disadvantage; diet at baseline (dietary intake of carotenoids and dietary intake of calcium); alcohol consumption at baseline; smoking status at baseline; adiposity at baseline; physical activity at F2; age at blood collection; and age at menopause. The identification, measurement and modelling of sociodemographic and lifestyle confounders are described in Online Resource 7. As age at menopause could only be measured for naturally postmenopausal women (821, 63% of the case-cohort after post hoc exclusions), this variable was not included in the adjustment set for the primary analyses. Sensitivity analyses were conducted, restricting to naturally postmenopausal women with a recorded age at menopause to include this variable in adjustment sets. Biomarkers that were identified as potential confounders *a priori* but had correlations ≥ 0.50 with the biomarker of interest were not included in the primary analysis (Online Resource 8).

The primary analyses modelled all biomarker concentrations as continuous, normalized values on the log_2_-scale. A one unit increase on the log_2_-scale represents a doubling in biomarker concentration. Analyses were repeated without adjustment for other biomarkers (where applicable). In addition, analyses that modelled concentrations of each sex-steroid hormone biomarker as quartiles corresponding to the distribution of normalized biomarker values in the subcohort were performed without adjustment for other biomarkers.

All analyses were complete-case analyses. The linearity assumption was tested for the continuous, normalized biomarker values using restricted cubic splines and Wald-tests. All statistical analyses were performed using Stata 16 (StataCorp, College Station, TX).

## 3. Results

Of the 1,312 women eligible after *post-hoc* exclusions, 87 were excluded due to missing sociodemographic and lifestyle confounder data (Fig. 1). In addition, 17 women were excluded due to missing measurements for all sex-steroid hormone biomarkers. The characteristics of the remaining 1,208 women are summarized in Table 1. Compared with non-cases, cases were more likely to be educated, have obesity, and experience the menopause at ≥ 53 years, and were less likely to be sufficiently active. The normalized concentrations of DHEA, total estradiol, free estradiol, leptin and CRP were higher, and the normalized concentration of SHBG was lower, for cases compared with non-cases. The characteristics of the 1,312 women eligible after *post-hoc* exclusions were not appreciably different from the 1,208 women analyzed (Online Resource 9).

### 3.1. Reliability of biomarker measurements and assay performance

The calculated overall intra-assay and inter-assay CVs were below 10% and 15% respectively for most biomarkers (Online Resource Table 4.1). The estimated intra-batch and inter-batch reliability ICCs were above 80% and 70% respectively for most biomarkers (Online Resource Table 4.2). The validity coefficients for the true and measured values of estradiol and testosterone were 0.987 and 0.997, respectively. Correlation plots are presented in Online Resource 5.

### 3.2. Risk ratios per doubling of biomarker concentration

For the primary analyses, increased risks of postmenopausal ER-positive breast cancer were observed per doubling plasma concentration of progesterone (RR: 1.22, 95% CI: 1.03 to 1.44), androstenedione (RR: 1.20, 95% CI: 0.99 to 1.45), DHEA (RR: 1.15, 95% CI: 1.00 to 1.34), total testosterone (RR: 1.11, 95% CI: 0.96 to 1.29), calculated free testosterone (RR: 1.12, 95% CI: 0.98 to 1.28), estrone (RR: 1.21, 95% CI: 0.99 to 1.48), total estradiol (RR: 1.19, 95% CI: 1.02 to 1.39) and calculated free estradiol (RR: 1.22, 95% CI: 1.05 to 1.41) (Table 2). A decreased risk was suggested for SHBG (RR: 0.83, 95% CI: 0.66 to 1.05).

Results did not appreciably differ in analyses without adjustment for other biomarkers (Table 2), except that the inverse association for SHBG was somewhat weaker (RR: 0.90, 95% CI: 0.73 to 1.11). For the sensitivity analyses in the subset of naturally postmenopausal women with a recorded age at menopause (Online Resource 10), the point estimates for RR were closer to the null for progesterone (RR: 1.11, 95% CI: 0.90 to 1.36) and androstenedione (RR: 1.08, 95% CI: 0.85 to 1.39), and further away from the null for estrone (RR: 1.30, 95% CI: 0.99 to 1.69), total estradiol (RR: 1.29, 95% CI: 1.04 to 1.58) and calculated free estradiol (RR: 1.31, 95% CI: 1.08 to 1.60). Results with and without adjustment for age at menopause were similar, whereas the point estimates for RR without adjustment for other biomarkers were closer to the null for estrone, free estradiol and SHBG (Online Resource 10).

### 3.3. Risk ratios for quartiles of biomarker concentration

The highest versus lowest levels of biomarker concentrations were associated with increased risks of postmenopausal ER-positive breast cancer for progesterone (RR: 1.56, 95% CI: 1.09 to 2.24), androstenedione (RR: 1.39, 95% CI: 0.97 to 2.00), DHEA (RR: 1.55, 95% CI: 1.06 to 2.25), total estradiol (RR: 1.49, 95% CI: 1.01 to 2.19) and calculated free estradiol (RR: 1.47, 95% CI: 0.99 to 2.17) (Table 3). RRs were suggestive of monotonic increases for DHEA, estrone and total estradiol. In contrast, the positive relationship between calculated free estradiol and postmenopausal ER-positive breast cancer plateaued at the third-highest plasma concentration compared to the lowest.

## 4. Discussion

Higher plasma concentrations of progesterone, estrogens and androgens, and decreasing plasma concentration of SHBG, were associated with increased risks of postmenopausal ER-positive breast cancer in this case-cohort of postmenopausal women. Similar results were obtained with and without control for other biomarkers that were identified as potential confounders, suggesting that confounding by the insulin/IGF-signaling and inflammatory pathways was minimal. The exception was SHBG; a somewhat stronger inverse relationship was observed with adjustment for adiponectin, leptin, insulin and IGF-1. Results of the sensitivity analyses in the subset of naturally postmenopausal women with a recorded age at menopause were not sensitive to adjustment for age at menopause. Rather, the deviations observed from the primary analyses could be explained by reduced precision in the subsample, or differences between women who were naturally postmenopausal (with a known age at menopause) and women who were assumed to be postmenopausal for other reasons.

A strength of our study was that careful consideration was given to biomarkers from the insulin/IGF-signaling and inflammatory pathways that may confound the associations between biomarkers of the sex-steroid hormone pathway and risk of postmenopausal ER-positive breast cancer. Biomarkers that may be potential confounders were identified *a priori* using a causal diagram that was informed by literature review and expert opinion. Causal diagrams can minimize the pitfalls of other confounder selection methods, including overadjustment bias [23, 39, 40]. However, residual confounding may remain if our assumptions are inaccurate or if important confounders have not been identified or correctly measured [39, 40]. Depicting the true complexity of biomarker interrelationships and their role in breast carcinogenesis is challenging. The current body of causal knowledge is limited, and we could not account for bidirectional relationships as the biomarkers had only been measured at one point in time. Thus, we assumed what the net direction of the effects of the measured biomarkers would be in a relatively older cohort of postmenopausal women in our causal diagram. Our assumptions can be refined with the advancement of causal knowledge over time, ideally in studies that measure biomarkers at multiple points in time.

The validity of our results depends upon the extent to which the measurements of the chosen biomarkers accurately represent the biological components of the inflammation, insulin/IGF-signaling and sex-steroid hormone pathways implicated in breast carcinogenesis. A major strength of our study was the use of a highly sensitive liquid chromatography-mass spectrometry method to measure the plasma concentrations of sex-steroid hormones in postmenopausal women with high precision and accuracy. We were able to demonstrate the validity of this method using reference standards for estradiol and testosterone. The measured and true values of estradiol and testosterone were highly correlated. Further, intra-assay and inter-assay CVs, as well as intra-batch and inter-batch ICCs, calculated from quality control samples indicated that the biomarker measurements were reliable, with few exceptions that may be attributable to batch and dispatch effects (Online Resource 4). We adopted a novel analysis approach to correct for batch effects, dispatch effects and time since last meal, whilst retaining meaningful biological variation in the biomarker measurements [33]. Further, we measured the plasma concentrations of a breadth of biomarkers selected through expert consultation and literature review. However, plasma concentrations of biomarkers measured at only one point in time will not be perfect proxies of complex and time-varying biological processes that may operate at cellular and systemic levels.

Our findings were generally consistent with previous studies, including a recent systematic review by Drummond et al. [4], a previous case-cohort study conducted at baseline (1990–1994) within the MCCS [13], and a pooled analysis of nine prospective studies examining the relationship between endogenous sex-steroid hormones and postmenopausal breast cancer [3]. A notable finding was the estimated risk ratio per doubling plasma concentration of progesterone; we observed the largest increased risk of postmenopausal ER-positive breast cancer for this biomarker (RR: 1.22, 95% CI: 1.03 to 1.44) compared to any other measured biomarker from the sex-steroid hormone pathway. Previous studies have either not measured endogenous progesterone or have drawn inconclusive results regarding its relationship with breast cancer after the menopause, largely due to insufficient assay sensitivity and low circulating levels in postmenopausal women [41]. Our result is in support of a recent study by Trabert at al. [42], which also used a highly sensitive liquid chromatography-mass spectrometry method and found increased risks of postmenopausal breast cancer per standard deviation increase in circulating endogenous progesterone levels (hazard ratio for invasive breast cancers: 1.24, 95% CI: 1.07 to 1.43). Trabert et al. [42] also present evidence for effect modification: reduced risks of postmenopausal breast cancer were observed with higher levels of progesterone among women in the lowest quintile of circulating estradiol (< 6.30 pg/mL), while increased risks were observed among women in the higher quintiles (≥ 6.30 pg/mL). Collectively, these results may challenge the plausibility of our *a priori* assumption that progesterone does not have a direct effect on postmenopausal ER-positive breast cancer (depicted by no direct arrow from progesterone to postmenopausal breast cancer in our causal diagram, Online Resource Fig. 8.1). This assumption was based on the systematic review by Drummond et al. [4], which found moderate quality evidence of no association between progesterone and breast cancer risk (albeit in both pre- and postmenopausal women combined). The implication of this assumption is that we should interpret the risk ratio for progesterone as an indirect effect, possibly driven by its role as a precursor of androgens and estrogens in steroidogenesis. This finding – in addition to concerns over the sensitivity of progesterone measurements in early studies, as well as studies demonstrating paracrine effects of progesterone via neighboring PR-positive cells [41] – warrants future studies including mediation analyses to determine what dictates the effect of progesterone on postmenopausal ER-positive breast cancer.

Our study confirms the causal role that sex-steroid hormones and SHBG play in the etiology of postmenopausal ER-positive breast cancer. We strengthen the causal evidence by demonstrating that potential confounding from other biological pathways implicated in breast carcinogenesis is likely non-substantial. Of note, two recent systematic reviews found insufficient evidence to establish a causal link between the inflammation and insulin/IGF-signaling pathways and breast cancer [8, 11]. Future research could examine whether adjustment for biomarkers from other biological pathways is more important for pre-menopausal breast cancer or ER-negative postmenopausal breast cancer. In addition, time-varying confounding could be examined in future studies that measure biomarkers at multiple points in time.

## Figures and Tables

**Table 1:** Characteristics of the analyzed case-cohort (N = 1,208)

	Cases N = 342		Non-Cases N = 866	
**Age at Blood Collection** (Years; Median, IQR)	66.0	(60.0, 71.0)	67.5	(61.0, 73.0)
**Dietary Calcium Intake** (mg/d; Median, IQR)	802.1	(621.1, 1045.8)	823.4	(610.2, 1051.8)
**Total Carotenoid Intake from Diet** (mcg/d; Median, IQR)	17885	(13726, 23441)	17274	(13352, 23188)
**Southern European Migrant Status** (N, %)				
No	277	81.0%	696	80.4%
Yes	65	19.0%	170	19.6%
**Socioeconomic Disadvantage** (N, %)				
Quintile 1: Most Disadvantaged	51	14.9%	134	15.5%
Quintile 2	56	16.4%	177	20.4%
Quintile 3	56	16.4%	127	14.7%
Quintile 4	72	21.1%	165	19.1%
Quintile 5: Least Disadvantaged	107	31.3%	263	30.4%
**Education** (N, %)				
Primary School or Some High / Technical School	202	59.1%	563	65.0%
Completed High / Technical School	65	19.0%	127	14.7%
Completed Tertiary Degree / Diploma	75	21.9%	176	20.3%
**Smoking Status** (N, %)				
Never Smoked	251	73.4%	637	73.6%
Ever Smoked	91	26.6%	229	26.4%
**Lifetime Alcohol Consumption** (N, %)				
Life Abstention	135	39.5%	318	36.7%
≤ 19 g/d	187	54.7%	493	56.9%
20 to 29 g/d	12	3.5%	26	3.0%
30 to 39 g/d	5	1.5%	17	2.0%
≥ 40 g/d	3	0.9%	12	1.4%
**Body Mass Index** (N, %)				
Normal (≥ 18.5 to < 25 kg/m^2^)	144	42.1%	374	43.2%
Overweight (≥ 25 to < 30 kg/m^2^)	110	32.2%	315	36.4%
Obese (≥ 30 kg/m^2^)	88	25.7%	177	20.4%
**Physical Activity**^a^ **(**N, %)				
Insufficiently Active	113	33.0%	266	30.7%
Sufficiently Active	80	23.4%	245	28.3%
Highly Active	149	43.6%	355	41.0%
**Age at Menopause**^b^ (N, %)				
≤ 48 years	41	20.8%	139	24.9%
49–50 years	53	26.9%	144	25.8%
51–52 years	41	20.8%	127	22.7%
≥ 53 years	62	31.5%	149	26.7%
**Normalized Biomarkers** (Median, IQR)				
**Sex-Steroid Hormone Pathway**				
Progesterone (nmol/L)	0.13	(0.10, 0.19)	0.13	(0.10, 0.17)
Androstenedione (nmol/L)	1.6	(1.2, 2.2)	1.5	(1.2, 2.0)
DHEA (nmol/L)	5.0	(3.3, 7.4)	4.5	(2.9, 6.8)
Estrone (pmol/L)	81.3	(60.2, 114.8)	78.7	(58.9, 107.5)
SHBG (nmol/L)	55.7	(41.6, 79.5)	61.9	(45.6, 82.3)
Total Testosterone (nmol/L)	0.64	(0.45, 0.87)	0.64	(0.44, 0.89)
Total Estradiol (pmol/L)	18.5	(12.5, 27.7)	16.3	(10.9, 25.1)
Free Testosterone (pmol/L)	5.5	(3.8, 8.4)	5.2	(3.6, 7.5)
Free Estradiol (pmol/L)	0.25	(0.15, 0.40)	0.20	(0.13, 0.33)
**Insulin/IGF-Signaling Pathway**				
Insulin (pg/mL)	298.0	(207.5, 422.7)	290.3	(208.7, 438.6)
IGF-1 (nmol/L)	7.8	(6.4, 9.4)	8.0	(6.4, 10.0)
IGFBP-3 (nmol/L)	66.2	(58.0, 75.5)	68.2	(58.8, 76.8)
C-Peptide (ng/mL)	2.6	(2.1, 3.4)	2.6	(2.0, 3.4)
**Inflammatory Pathway**				
Leptin (pg/mL)	16312	(8441, 31909)	14056	(6387, 27578)
Adiponectin (ng/mL)	25514	(19623, 32838)	24912	(18922, 33098)
TNF-α (pg/mL)	2.7	(2.2, 3.3)	2.6	(2.2, 3.2)
IL-6 (pg/mL)	0.73	(0.55, 1.04)	0.73	(0.52, 1.02)
IL-8 (pg/mL)	2.8	(2.3, 3.9)	3.0	(2.2, 4.0)
IL-10 (pg/mL)	0.25	(0.19, 0.33)	0.23	(0.17, 0.32)
IFN-γ (pg/mL)	5.5	(4.1, 7.8)	5.4	(3.8, 8.6)
CRP (ng/mL)	1633	(804, 2936)	1391	(682, 3020)

N: Number. IQR: Interquartile range. DHEA: Dehydroepiandrosterone. SHBG: Sex-hormone binding globulin. IGF: Insulin-like growth factor. IGF-1: Insulin-like growth factor-1. IGFBP-3: Insulin-like growth factor binding protein-3. TNF-α: Tumor necrosis growth factor-alpha. IL-6: Interleukin-6. IL-8: Interleukin-8. IL-10: Interleukin-10. IFN-γ: Interferon gamma. CRP: C-reactive protein. nmol/L: Nanomoles per liter. pmol/L: Picomoles per liter. ng/mL: Nanograms per milliliter. pg/mL: Picograms per milliliter. g/d: Grams per day. mg/d: Milligrams per day. mcg/d: Micrograms per day. kg/m^2^: Kilograms per meters squared.

a Physical activity was measured as total weighted minutes of walking, moderate- and vigorous-intensity recreation- and transport-related physical activity (MVPA) per week at the second follow-up wave. Insufficiently active was defined as < 150 total weighted minutes of MVPA per week, sufficiently active was defined as 150 to ≤ 300 total weighted minutes of MVPA per week, and highly active was defined as > 300 total weighted minutes of MVPA per week.

b Age at menopause was measured for naturally postmenopausal women only, when the cessation of periods for 12 months was first documented (baseline, the first follow-up wave, or the second follow-up wave).

Missing data for normalized biomarkers are as follows: 1 for progesterone; 2 for estrone; 8 for estradiol; 1 for adiponectin; 4 for CRP. Missing data for other covariates include: 452 for age at menopause (including 49 naturally postmenopausal women).

Southern European Migrant status, socioeconomic disadvantage, education, smoking status, lifetime alcohol consumption, body mass index, dietary calcium intake and total carotenoid intake from diet were measured at baseline. Biomarker concentrations, age at blood collection and physical activity were measured at the second follow-up wave.

**Table 2:** Risk ratios for postmenopausal estrogen receptor-positive breast cancer per doubling of biomarker concentration

Biomarker (per doubling concentration)	Cases	Subcohort Non-Cases	Risk Ratio	95% CI
**Progesterone (nmol/L)**				
Primary analysis	342	865	1.22	(1.03, 1.44)
**Androstenedione (nmol/L)**				
Primary analysis	342	866	1.20	(0.99, 1.45)
**DHEA (nmol/L)**				
Primary analysis	342	866	1.15	(1.00, 1.34)
**Total Testosterone (nmol/L)**				
Primary analysis (adjusted for SHBG)	342	866	1.11	(0.96, 1.29)
Not adjusted for other biomarkers	342	866	1.10	(0.95, 1.27)
**Free Testosterone (nmol/L)**				
Primary analysis	342	866	1.12	(0.98, 1.28)
**Estrone (pmol/L)**				
Primary analysis (adjusted for adiponectin, leptin, TNF-α, IL-6, insulin, IGF-1 and SHBG)	342	863	1.21	(0.99, 1.48)
Not adjusted for other biomarkers	342	864	1.20	(0.98, 1.45)
**Total Estradiol (pmol/L)**				
Primary analysis (adjusted for adiponectin, leptin, TNF-α, IL-6, insulin, IGF-1 and SHBG)	341	858	1.19	(1.02, 1.39)
Not adjusted for other biomarkers	341	859	1.20	(1.04, 1.38)
**Free Estradiol (pmol/L)**				
Primary analysis (adjusted for adiponectin, leptin, TNF-α, IL-6, insulin and IGF-1)	341	858	1.22	(1.05, 1.41)
Not adjusted for other biomarkers	341	859	1.18	(1.03, 1.35)
**SHBG (nmol/L)**				
Primary analysis (adjusted for adiponectin, leptin, insulin and IGF-1)	342	865	0.83	(0.66, 1.05)
Not adjusted for other biomarkers	342	866	0.90	(0.73, 1.11)

CI: Confidence interval. DHEA: Dehydroepiandrosterone. SHBG: Sex hormone binding globulin. IGF-1: Insulin-like growth factor-1. IL-6: Interleukin-6. TNF-α: Tumor necrosis factor-alpha. nmol/L: Nanomoles per liter. pmol/L: Picomoles per liter.

The results of the primary analyses were adjusted for sociodemographic and lifestyle confounders (education, socioeconomic disadvantage, Southern European Migrant status, dietary intake of carotenoids at baseline, dietary intake of calcium at baseline, lifestyle alcohol consumption at baseline, smoking status at baseline, adiposity at baseline, physical activity at the second follow-up wave and age at blood collection) and other biomarkers identified as potential confounders, where applicable (Online Resource 8).

**Table 3:** Risk ratios for postmenopausal estrogen receptor-positive breast cancer, by quartiles of biomarker concentrations

Quartiles^a^ of Normalized Biomarker Concentrations	Cases	Subcohort Non-Cases	Risk Ratio	95% CI
**Progesterone**				
Quartile 1	72	208	Ref	Ref
Quartile 2	92	218	1.25	(0.87, 1.81)
Quartile 3	67	220	0.96	(0.65, 1.41)
Quartile 4	111	219	1.56	(1.09, 2.24)
**Androstenedione**				
Quartile 1	72	214	Ref	Ref
Quartile 2	97	214	1.32	(0.92, 1.90)
Quartile 3	69	219	0.92	(0.63, 1.35)
Quartile 4	104	219	1.39	(0.97, 2.00)
**DHEA**				
Quartile 1	65	216	Ref	Ref
Quartile 2	75	207	1.19	(0.82, 1.74)
Quartile 3	94	224	1.38	(0.94, 2.00)
Quartile 4	108	219	1.55	(1.06, 2.25)
**Total Testosterone**				
Quartile 1	80	211	Ref	Ref
Quartile 2	85	216	1.04	(0.73, 1.49)
Quartile 3	98	223	1.16	(0.82, 1.65)
Quartile 4	79	216	1.04	(0.72, 1.50)
**Free Testosterone**				
Quartile 1	75	218	Ref	Ref
Quartile 2	78	203	1.08	(0.75, 1.55)
Quartile 3	83	225	1.02	(0.71, 1.47)
Quartile 3	106	220	1.26	(0.89, 1.80)
**Estrone**				
Quartile 1	79	207	Ref	Ref
Quartile 2	78	214	0.95	(0.66, 1.37)
Quartile 3	86	223	1.03	(0.72, 1.48)
Quartile 4	99	220	1.13	(0.78, 1.64)
**Total Estradiol**				
Quartile 1	64	215	Ref	Ref
Quartile 2	81	212	1.27	(0.87, 1.85)
Quartile 3	93	218	1.41	(0.97, 2.05)
Quartile 4	103	214	1.49	(1.01, 2.19)
**Free Estradiol**				
Quartile 1	67	216	Ref	Ref
Quartile 2	64	214	0.95	(0.64, 1.41)
Quartile 3	101	213	1.46	(1.01, 2.12)
Quartile 4	109	216	1.47	(0.99, 2.17)
**SHBG**				
Quartile 1	112	215	Ref	Ref
Quartile 2	83	213	0.79	(0.56, 1.10)
Quartile 3	72	224	0.67	(0.47, 0.95)
Quartile 4	75	214	0.83	(0.57, 1.21)

CI: Confidence interval. Ref: Reference category. DHEA: Dehydroepiandrosterone. SHBG: Sex-hormone binding globulin.

a Quartiles based on the distribution of normalized biomarker values in the subcohort. Minimum, median and maximum values for each quartile are presented in Online Resource 11.

Results were adjusted for sociodemographic and lifestyle confounders (education, socioeconomic disadvantage, Southern European Migrant status, dietary intake of carotenoids at baseline, dietary intake of calcium at baseline, lifestyle alcohol consumption at baseline, smoking status at baseline, adiposity at baseline, physical activity at the second follow-up wave and age at blood collection).

## Data Availability

The dataset generated for the current study is not publicly available due to compliance with participant informed consent and human research ethics committee approvals, but can be requested by contacting pedigree@cancervic.org.au.
